# Sympathetic transmitters control thermogenic efficacy of brown adipocytes by modulating mitochondrial complex V

**DOI:** 10.1038/sigtrans.2017.60

**Published:** 2017-11-10

**Authors:** Tao-Rong Xie, Chun-Feng Liu, Jian-Sheng Kang

**Affiliations:** 1 CAS Key Laboratory of Nutrition and Metabolism, Institute for Nutritional Sciences, Shanghai Institutes for Biological Sciences, Chinese Academy of Sciences, Shanghai, China; 2 Technical Center for Animal, Plant and Food Inspection and Quarantine, Shanghai Entry-Exit Inspection and Quarantine Bureau, Shanghai, China

## Abstract

Obesity is a worldwide epidemic and results from excessive energy intake or inefficient energy expenditure. It is promising to utilize the thermogenic function of brown adipose tissue for obesity intervention. However, the mechanisms controlling the efficacy of norepinephrine-induced thermogenesis in brown adipocytes remain elusive. Here we demonstrate that norepinephrine (NE) induces low-efficacy thermogenesis, evoking both heterogeneous changes (ΔΨ_m_ and ΔpH) and homogenous responses, one of which is that NE stimulation causes large amounts of ATP consumption in brown adipocytes. We reveal that the proton-ATPase activity of mitochondrial complex V is a key factor that antagonizes proton leakage by UCP1 and determines the efficacy of NE-induced thermogenesis in brown adipocytes. Furthermore, to avoid unnecessary and undesired heat production, we reveal that ATP is a necessary sympathetic cotransmitter for the high efficacy and specificity of NE-induced thermogenesis in brown adipocytes as it increases intracellular calcium concentrations and upregulates the ATP synthase activity of complex V. Thus, we demonstrate the modulation mechanism of thermogenic efficacy in brown adipocytes. These findings imply new strategies to partially or fully utilize the thermogenic capacity of brown adipocytes to identify therapeutic targets for the treatment of obesity and diabetes.

## Introduction

Obesity is a worldwide epidemic and results from excessive energy intake or inefficient energy expenditure. Brown adipose tissue (BAT) is the major tissue for cold-induced thermogenesis (energy dissipation as heat) without shivering.^1,2^ The identification of functional BAT in adult humans^3–5^ suggests that it is promising to utilize the thermogenic capacity of BAT for obesity and diabetes treatments.^6,7^

Currently, the mechanism of thermogenesis in BAT and brown adipocytes (BA) is known in general.^2^ Cutaneous thermosensory signals for cold stimulation evoke sympathetic nerve firing in BAT via the hypothalamus.^8^ The released neurotransmitter norepinephrine (NE) binds the adrenoreceptors of BA, upregulates hormone-sensitive lipase (HSL)-mediated lipolysis via phosphorylation by protein kinase A signaling, mobilizes free fatty acids to activate mitochondrial uncoupling protein-1 (UCP1) and converts electrochemical potential energy stored in the mitochondrial proton gradient to heat.^2,9^ However, our previous study demonstrates the low thermogenic efficacy of NE-induced thermogenesis in BA,^10^ which may partially account for the failures of trials utilizing the thermogenic capacity of BAT for obesity intervention.^11,12^ Therefore, the regulatory mechanism of thermogenesis in BA is still largely unknown. In this study, we demonstrate that the proton-ATPase function of mitochondrial complex V accounts for NE-induced heterogeneous changes in BA and that sympathetic cotransmitter ATP enhances the efficacy of thermogenesis in BA.

## Materials and methods

**Figure 3:**
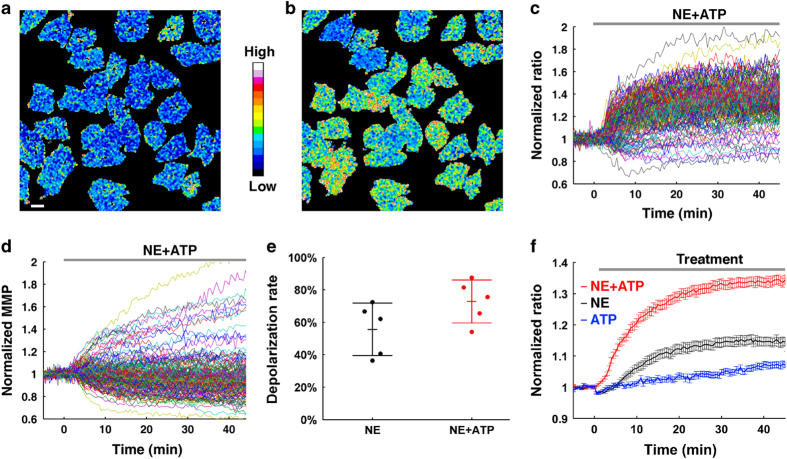
Sympathetic cotransmitter ATP enhances NE-induced thermogenic efficacy in BA. (**a** and **b**) Representative thermal images of BA before and after 0.1 μM NE and 10 μM ATP co-treatment, respectively. Scale bar, 20 μm. (**c**) 0.1 μM NE and 10 μM ATP-induced thermal responses in BA. Each colored trace represents the thermogenic response of a single BA (*n*=158). (**d**) The dynamic plots of MMP demonstrate that most BA show MMP depolarization after NE and ATP co-treatment. Each colored trace represents an MMP dynamic of a single BA (*n*=158). (**e**) Shows scatter plots of MMP depolarization percentage in 0.1 μM NE without (black) or with (red) ATP-treated experiments. NE and ATP co-treatment increases the percentage of MMP depolarization (72.8±13.3%) compared to NE treatment alone (55.6±16.1%). Error bars in the figure represent mean±s.d. (**f**) 0.1 μM NE without (black line, also in Figure 1h) or with (red line, *n*=158) 10 μM ATP-induced thermogenesis in BA. The blue line shows the control results of ATP treatment alone in BA (*n*=67). All data points in **f** represent mean±s.e.m.

Data are pooled from or repeated for each condition with at least three independent experiments. No statistical methods were used to predetermine the sample size. All data points in the figures represent mean±s.e.m., except for Figure 3e in which data in the text or figures represent mean±s.d. More detailed information on the experimental procedures can be found in the Supplementary Data.

### Isolation and primary culture of brown adipocytes

BA were isolated from 3- to 4-week-old male C57BL/6J mice with a procedure similar to that described by Lucero *et al.*^13^ Briefly, mice were kept at 4 °C overnight with free access to food and water to deplete stored lipids in BAT. The mice were then killed by cervical dislocation and swabbed with 75% ethanol. Interscapular BAT was isolated and placed in an isolation buffer (DMEM with 4% NCS). The tissue was minced and digested with 0.2% collagenase type II in a shaking water bath at 37 °C for 30 min.^14^ After digestion, the reaction mixture was discarded and the tissue was washed with isolation buffer. Cells were dissociated by gently triturating with fire-polish pipettes and washed by centrifugation in PBS. After the final washing, the cells were plated onto 12-mm coverslips (~3×10^4^ cells per coverslip). Coverslips were precoated with Matrigel. After 2 h, 2 ml of plating medium (DMEM supplemented with 5% FBS, 100 units per ml penicillin and 100 μg ml^−1^ streptomycin) was added to each 35-mm dish. From the second day in culture onward, half of the medium was replaced with feeding medium (plating medium supplemented with Ara-C to inhibit fibroblast proliferation and 2 μM final concentration of Ara-C) every 2 days. Cells were maintained at 37 °C in a humidified atmosphere of 95% air and 5% CO_2_ and used for imaging at 3–8 days *in vitro*.

### Cell transfection

BA were washed with PBS and resuspended in electroporation solution (20 mM Hepes, 135 mM KCl, 2 mM MgCl_2_, 0.5% Ficoll 400, 1% DMSO, 2 mM ATP and 5 mM glutathione, pH 7.6) with 20 μg of plasmid DNA.^15^ Two pulses (115V, 10-ms duration) with 1-s intervals were delivered with an ECM 830 Square Wave Electroporation System (Harvard Apparatus, Inc., Holliston, MA, USA) to electroporate cells (1–1.5×10^6^ cells per ml). Then, cells were seeded onto Matrigel-coated coverslips. BA were maintained in DMEM supplemented with 5% FBS until imaging.

### Thermogenic study

All imaging was performed using a confocal microscope with a 40×/0.95 objective (Olympus, Shinjuku, Japan) for time-lapse imaging and a 100×/1.4O objective (Olympus) for high-resolution imaging. Cells were co-stained with RhB-ME and Rh800 (20 nM dyes for time-lapse imaging and 50 nM dyes for high-resolution imaging) in Tyrode’s solution (in mM: 10 Hepes, 10 glucose, 3 KCl, 145 NaCl, 1.2 CaCl_2_, and 1.2 MgCl_2_, pH 7.4) for 1 h at 33 °C.^10^ The pseudocolor of the RhB-ME channel is red (excited at 559 nm and collected at 575–620 nm), and the Rh800 channel is green (excited at 635 nm and collected at 655–755 nm). All images were collected at 512×512 (for time-lapse imaging) and 1600×1600 (for high-resolution imaging) pixel resolutions (12 bit).

To minimize the heat influence of the perfusion solution, time-lapse imaging of BA was performed in 2 ml of Tyrode’s solution rather than in the perfusion system. To minimize bleaching and damage to live BA in time-lapse imaging, the lowest intensity of lasers with the largest pinhole setting and the shortest scanning time were used, and 101 frames were recorded in time-lapse imaging at 30-s intervals. NE (0.1 μM), ATP (10 μM) or vehicle was injected as early as the 11th frame of time-lapse imaging for the thermogenesis studies in BA. SR-59230 A (1 μM) or oligomycin (10 μg ml^−1^) was injected 15 min before the time-lapse imaging in pharmacological tests.

After background was removed, ratiometric values of the Rh800 channel to the RhB-ME channel (simultaneously excited by 635-nm and 559-nm lasers) were calculated pixel by pixel to represent the thermal response of the sample. For noise reduction, the pixels with signal-to-noise ratios less than 1.5 were excluded, and a 5×5 moving average was used before the ratiometric process. According to the three-sigma rule, the outlier (99.7% tolerance interval) of the ratios was also excluded. The ratio of each cell was the average ratio of all pixels representing the cell. Since we focused on thermal responses, the mean ratio of the steady state before drug treatments was used to normalize every data point for each cell. All data analysis was performed with MATLAB (MathWorks Inc., Natick, MA, USA) and ImageJ (NIH, Bethesda, MD, USA).

### Cytoplasmic pH study

Cytoplasmic pH imaging was performed using a confocal microscope with a 40×/0.95 objective (Olympus) for time-lapse imaging. Cells were co-stained with 5 μM SNARF1-AM and 20 nM Rh800 in Tyrode’s solution for 30 min at 37 °C. Then, SNARF1-AM was washed out using Tyrode’s solution (with 20 nM Rh800). The pseudocolor of the acidification channel is red (excited by 561-nm laser and collected with 575–600-nm bandpass filter), the alkalization channel is green (simultaneously collected with 600–675-nm bandpass filter), and the Rh800 channel is magenta (sequentially excited by 635-nm laser and collected with 655–755-nm bandpass filter). Ratiometric values of the acidification channel to the alkalization channel were calculated pixel by pixel to represent the relative pH value of the sample. The other conditions of imaging, data acquisition and analysis for the cytoplasmic pH study were the same as the conditions in the thermogenic study.

### Redox (FAD/FAD+NADH) measurement

Redox (FAD/FAD+NADH) imaging was performed using a customized fluorescence microscope with a 40×/0.8 W objective (Olympus) for time-lapse imaging. The endogenous autofluorescence images^16^ were excited with an Optoscan monochromator (Cairn Research Ltd., Kent, UK). The pseudocolor of the FAD channel is red (excited by 430-nm light with 20-nm bandwidth and collected with 525–575-nm bandpass filter), whereas the NADH+FAD channel is green (sequentially excited by 340-nm light with 20-nm bandwidth and collected with 420-nm long pass filter). In addition, the fluorescence images were acquired with an Evolve 512 EMCCD (Photometrics Ltd., Tucson, UK). Time-lapse imaging of BA was performed in 4 ml of Tyrode’s solution at 33 °C. Sixty frames were recorded in a time-lapse imaging at 30-s intervals. NE (0.1 μM) was injected as early as the 11th frame of time-lapse imaging for the redox studies in BA. The other conditions of imaging, data acquisition and analysis for the redox study were the same as the conditions in the thermogenic study.

### Cytoplasmic [Ca^2+^] study

Cytoplasmic [Ca^2+^] imaging was performed using the customized fluorescence microscope with a 40×/0.8 W objective (Olympus) for time-lapse imaging and an Optoscan monochromator (Cairn Research Ltd.) as a light source. Cells were stained with 5 μM Fura2-AM in Tyrode’s solution for 30 min at 37 °C. Then, Fura2-AM was washed out using Tyrode’s solution. The pseudocolor of the 380-nm channel is red (excited by 380-nm light with 10-nm bandwidth and collected with 505–535-nm bandpass filter), and the 340-nm channel is green (sequentially excited by 340-nm light with 10-nm bandwidth and collected with 505–535-nm bandpass filter). Ratiometric values of the 340-nm channel to the 380-nm channel were calculated pixel by pixel to represent the relative [Ca^2+^] of the sample. The other conditions of imaging, data acquisition and analysis for the cytoplasmic [Ca^2+^] study were the same as the conditions in the redox study.

### Mitochondrial [Ca^2+^] study

Mitochondrial [Ca^2+^] imaging was performed using a confocal microscope with a 40×/0.95 objective (Olympus) for time-lapse imaging. Cells were transfected with 4mtD3cpv (from Dr R Tsien) using an ECM 830 Square Wave Electroporation System (Harvard Apparatus, Inc., USA). The pseudocolor of the CFP channel is cyan (excited by 440-nm laser and collected with 480–495-nm bandwidth filter) and the YFP channel is magenta (simultaneously collected with 505–605-nm bandwidth filter). Ratiometric values of the YFP channel to the CFP channel were calculated pixel by pixel to represent the relative [Ca^2+^] of the sample. The other conditions of imaging, data acquisition and analysis for the mitochondrial [Ca^2+^] study were the same as the conditions in the cytoplasmic [Ca^2+^] study.

### Cytoplasmic and mitochondrial [ATP] study

[ATP] imaging was performed using a confocal microscope with a 40×/0.95 objective (Olympus) for time-lapse imaging. Cells were transfected with AT1.03 for cytoplasmic [ATP] or mitAT1.03 for mitochondrial [ATP] (from Dr H Noji and Dr H Imamura) using an ECM 830 Square Wave Electroporation System (Harvard Apparatus, Inc.). The pseudocolor of the CFP channel is cyan (excited at 440 nm and collected at 480–495 nm), and the YFP channel is magenta (simultaneously excited at 440 nm and collected at 505–605 nm). Ratiometric values of the YFP channel to the CFP channel were calculated pixel by pixel to represent the relative [ATP] of the sample. The other conditions of imaging, data acquisition and analysis for the [ATP] study were the same as the conditions in the thermogenic study.

## Results

### NE induces heterogeneous responses in BA

**Figure 1:**
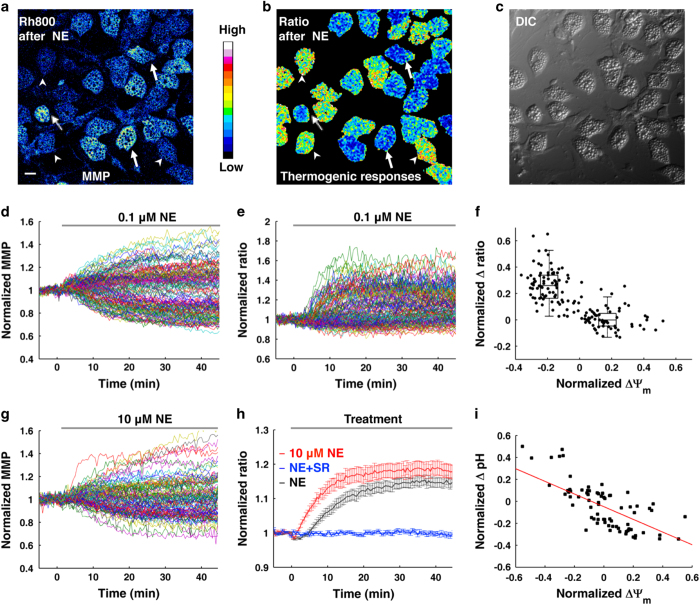
NE induces heterogeneous responses in BA. (**a**–**f**) NE-induced thermogenic responses in BA show a negative correlation with ΔΨ_m_. (**a**) Representative MMP image of BA after 0.1 μM NE treatment. Arrow heads point to BA with mitochondrial depolarization, whereas arrows point to BA with mitochondrial hyperpolarization. Scale bar, 20 μm. (**b**) The corresponding thermal image (ratio of Rh800 intensity to RhB-ME intensity) after 0.1 μM NE treatment. The result shows the low efficacy of NE-induced thermogenic responses in BA. Some BA show thermogenic responses to NE (arrow heads), whereas others have no response to NE stimulation (arrows). (**c**) Differential interference contrast (DIC) image of BA. (**d**) The raw data plots of MMP show subpopulations of depolarization and hyperpolarization in 0.1 μM NE-treated BA. Each colored trace represents a MMP change of a single BA (*n*=150). (**e**) NE-induced thermal responses in BA. Each colored trace represents a thermogenic response of a single BA (*n*=150). (**f**) The scatter plot of thermogenic responses (**d**) versus MMP changes (**e**) induced by NE, which shows a negative correlation (*r*=−0.73, *n*=150, and *P*=2.97×10^−26^ by Pearson’s correlation coefficient test). The box plots of the depolarization subpopulation and the hyperpolarization subpopulation show significantly different amplitudes of thermogenic responses (*P*=1.88×10^−27^, two-sided *t*-test). (**g**) The raw data plots of MMP still show two-subpopulations in 10 μM NE-treated BA. Each colored trace represents an MMP change of a single BA (*n*=102). (**h**) Thermogenic responses in BA are evoked by 0.1 μM NE (black line, *n*=150) and 10 μM NE (red line, *n*=88), respectively. The blue line shows that the NE-induced thermogenic response of BA is inhibited by 1 μM SR-59230 A (*n*=97). All data points in figures represent mean±s.e.m. (**i**) A representative scatter plot of pH changes versus MMP changes induced by 0.1 μM NE, which shows a strong negative correlation (red line, *r*=-0.67, *n*=75, and *P*=3.94×10^−11^ by Pearson’s correlation coefficient test).

We consistently observed that NE-induced heterogeneous changes in mitochondrial membrane potentials (MMP) in BA (Figure 1), which was monitored with thermoneutral rhodamine 800 (Rh800, a thermal-insensitive mitochondrial marker and MMP sensor).^10,17^ Obviously, there are two MMP populations (depolarization and hyperpolarization) of NE-induced thermogenesis in BA (Figures 1a–h), which is in line with previous studies.^10,18^ The subpopulation of MMP depolarization can be explained by the activation of UCP1, which dissipates the electrochemical potential energy of protons as heat. The diverse extent of MMP depolarization evoked by NE may be explained by the heterogeneity and different NE affinities of the β adrenergic receptors^19^ or the heterogeneous levels of UCP1 expression.^20^

To test this explanation, we evaluated the potential relationship between the heterogeneity of MMP changes and the extent of HSL phosphorylation at Ser563 (Supplementary Figure 1), as HSL serves as a downstream lipase of β-adrenergic receptors. After NE stimulation and monitoring of the responses of MMP, we retrospectively immunostained BA with an antibody against phospho-HSL and assumed that BA with high levels of phosphorylated HSL should correlate with mitochondrial depolarization. However, BA with high levels of phosphorylated HSL (arrows and red arrow head) either show mitochondrial depolarization (arrow heads) or hyperpolarization (arrows), whereas BA with low levels of phosphorylated HSL (white arrow heads) can show mitochondrial depolarization (Supplementary Figures 1b, c and f). Second, treatments with 100-fold higher NE concentrations (10 μM) still showed two MMP populations (Figure 1g). These results suggest that the heterogeneous changes of MMP are unlikely to be correlated to the heterogeneity of adrenoreceptors or different activities of HSL. In addition, we also found that all BA express UCP1, but it is expressed at heterogeneous levels (Supplementary Figures 2a–d), and that mitochondrial hyperpolarization is not due to the absence of UCP1 (Supplementary Figures 2e–g). These results suggest that the heterogeneities of p-HSL or UCP1 may only partially account for the diverse responses of mitochondrial MMP.

### Correlations among NE-induced heterogeneous responses in BA

With thermosensitive rhodamine B methyl ester (RhB-ME)-based mito-thermometry,^10^ we observed that the NE-induced thermogenic responses (the fluorescent intensity ratio of Rh800 to RhB-ME) in BA indeed show a negative correlation with mitochondrial electric potential difference (ΔΨ_m_) (Figures 1d–f and Supplementary Movies 1–2). Our findings suggest that the heterogeneous responses of MMP in BA can index NE-induced thermogenic efficacy, and they further confirm that the efficacy of NE-induced thermogenesis is low.^10^ Particularly, even a high concentration (10 μM) of NE still shows low efficacy with two ΔΨ_m_ populations (Figure 1g) and only slightly increases the thermogenic responses of BA compared to stimulation with 0.1 μM NE (Figure 1h). However, the NE-induced thermogenic responses of BA are inhibited by 1 μM SR-59230A (a potent β-adrenergic receptor antagonist, Figure 1h).

Considering that mitochondrial ΔΨ_m_ is quasi-linear in physiologically relevant ranges of pH difference (ΔpH),^21^ MMP hyperpolarization might result from cytoplasmic acidification by enhanced glycolysis or NE-stimulated activities of proton pumps. Indeed, using SNARF1-AM, a ratiometric pH-indicating fluorescent probe, we revealed that the subpopulation of MMP hyperpolarization shows cytoplasmic acidification (ΔpH<0) and that ΔΨ_m_ is negatively correlated with ΔpH (Figure 1i).

In addition, the phosphorylation level of HSL consistently shows little correlation (Supplementary Figure 3a), whereas the heterogeneity of UCP1 shows a reasonable positive correlation (Supplementary Figure 3b) with NE-induced thermogenesis in BA. Intriguingly, the correlations among NE-induced thermogenic responses, ΔΨ_m_, ΔpH (strong negative correlations, Figures 1f and i) and UCP1 (moderate positive correlation) suggest that the mitochondrial thermogenesis of BA might be the net result of proton outflow by pumps and proton leakage by UCP1.

### NE stimulation activates the proton-ATPase activity of mitochondrial complex V in BA

**Figure 2:**
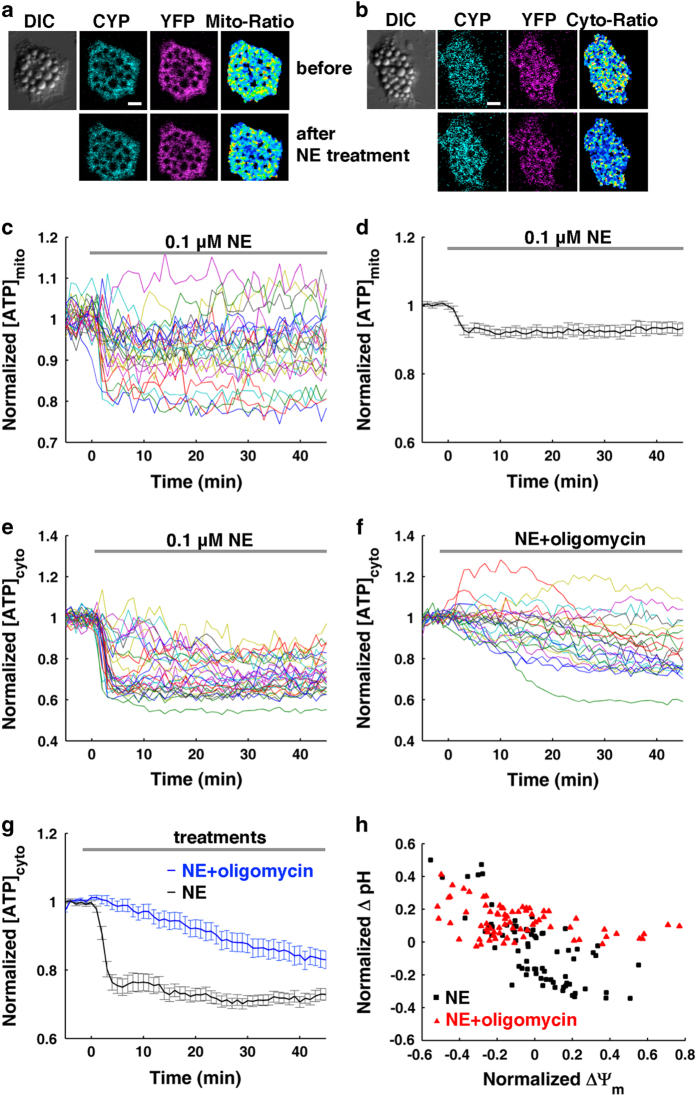
NE stimulates proton-ATPase activity of mitochondrial complex V in BA. (**a**–**g**) Fluorescence of AT1.03-based ratiometric measurements of mitochondrial and cytoplasmic ATP. (**a** and **b**) Show fluorescence images of mitAT1.03- and AT1.03-transfected BA before and after 0.1 μM NE treatment, respectively. Scale bar, 20 μm. (**c** and **d**) Show raw data plots (**c**) and an average plot (**d**) of mitochondrial ATP changes (*n*=16) in 0.1 μM NE-treated BA. Each colored trace represents the ATP change of a single BA (**c**). (**e**) Shows the raw data plots of cytoplasmic ATP (*n*=29) after NE treatment. Each colored trace represents the ATP change of a single BA. (**f**) Shows the raw data plots of cytoplasmic ATP (*n*=23) after co-treatment of NE and oligomycin A. Each colored trace represents the ATP change of a single BA. (**g**) Compares the averaged changes in cytoplasmic ATP after NE treatment (*n*=29) or NE and oligomycin A co-treatment (*n*=23), which show that the inhibitor (oligomycin A) of mitochondrial complex V mitigates the ATP consumption induced by NE stimulation. (**h**) Shows a representative scatter plot of pH changes versus MMP changes induced by 0.1 μM NE with (red, *n*=75) or without (black, *n*=75) 10 μg ml^−1^ oligomycin A pretreatment, which demonstrates that the inhibitor of mitochondrial complex V blocks the activity of the proton pump. All data points in **d** and **g** represent mean±s.e.m.

Cytoplasmic acidification of MMP-hyperpolarized BA (Figure 1i) can be not only explained by NE-activated proton ATPase (H^+^-ATPase), but also by enhanced glycolysis. To distinguish the difference and identify the potential roles of enhanced glycolysis (net ATP production) or NE-activated mitochondrial H^+^-ATPase (ATP consumption) in BA, we next examined the statuses of mitochondrial and cytoplasmic ATP concentrations ([ATP]) after NE stimulation (Figures 2a–g). Using FRET-based ATP indicators (mitAT1.03 and AT1.03),^22^ we found that both cytoplasmic [ATP] and the majority of mitochondrial [ATP] are decreased (Figures 2c and e) after NE stimulation of BA.

Clearly, these results suggest that NE stimulation activates an ATPase, which is likely a H^+^-ATPase, that might play a role in the thermogenic efficacy of BA. The best candidate for the H^+^-ATPase is mitochondrial ATP synthase/complex V, which can function as an ATPase *in vitro* to pump protons.^23^ ATP is consumed by not only mitochondrial ATPase, but also other cellular activities. Therefore, it was necessary to further verify whether complex V is the ATPase activated by NE. Thus, we used oligomycin A, a specific inhibitor of complex V, and revealed that oligomycin A mitigates the ATP consumption induced by NE stimulation (Figures 2f and g). These results confirmed that NE activates the ATPase function of mitochondrial complex V, which consumes large amounts of ATP assimilating from the cytosol. For complex V-independent ATP consumption, other ATPases, such as adenylate cyclase, could offer an explanation. After NE binds β receptors and stimulates the activity of adenylate cyclase via Gs, adenylate cyclase then consumes ATP to produce cAMP, which is necessary for protein kinase A activation.

The role of mitochondrial complex V as an ATPase *in vivo* is quite provocative. For example, where does the ATP come from? The source of ATP production can be provided by NE-stimulated metabolism of glucose because it has been reported and is well known that glucose uptake is increased in BA or BAT by one or two orders of magnitude in human (12-fold) and rat (110 times) under cold stimulation.^24,25^ NE-stimulated metabolism in BA is indeed supported by accelerated metabolic status represented by homogenous redox changes (Supplementary Figures 4a–h), which are shown in endogenous autofluorescence images and the ratio measurement of FAD and NADH.^16^ In addition, as interestingly illustrated in Figure 2f, few BA pretreated with oligomycin A show transiently increased cytoplasmic [ATP], which supports accelerated cytosol metabolism for ATP production after NE stimulation. Moreover, accelerated metabolic status shows a strong positive correlation with NE-induced thermogenesis in BA (Supplementary Figure 5).

To further check whether the ATPase function of complex V causes cytoplasmic acidification after NE stimulation, we used oligomycin A to inhibit complex V again and revealed that almost all BA showed cytoplasmic alkalizations (ΔpH>0) and that most BA showed MMP depolarization after NE stimulation (Figure 2h and Supplementary Figure 6). Thus, these findings further demonstrated and confirmed that NE stimulation activates the H^+^-ATPase function of complex V instead of glycolysis for cytoplasmic acidification. Together, our findings suggest that the H^+^-ATPase activity of complex V is a key factor in antagonizing proton leakage by UCP1, which impacts the net cytoplasmic ΔpH (Figures 1i and 2h) and modulates the efficacy of NE-induced BA thermogenesis (Supplementary Figure 6b). The efficacy is comparable to CCCP-induced thermogenesis.^10^

### ATP as a sympathetic cotransmitter can increase NE-induced thermogenic efficacy

Our observations suggest that factors other than NE alone are needed for the high efficacy of evoking thermogenesis in BAT. Considering that ATP is also a neurotransmitter and is co-released with NE when sympathetic nerve firing is evoked,^26^ we examined whether ATP is needed as a co-factor for NE to efficiently evoke thermogenesis in BA (Figure 3). As illustrated in Figure 3, most BA showed thermogenic responses (Figures 3a–c) to NE and ATP co-stimulation (Supplementary Movies 3–4). The co-treatment with NE and ATP also increases the percentage (in mean±s.d.) of the MMP depolarization subpopulation (72.8±13.3%) in BA compared to NE treatment alone (55.6±16.1% and *P*=0.0514, one-sided *t*-test) (Figures 3d–e). NE and ATP co-stimulation markedly increased the amplitude of thermogenic responses (Figure 3f) in BA compared to NE treatment alone (Supplementary Movies 1–4). In contrast, BA only showed slightly and relative flat responses to ATP treatment alone (Figure 3f).

**Figure 4:**
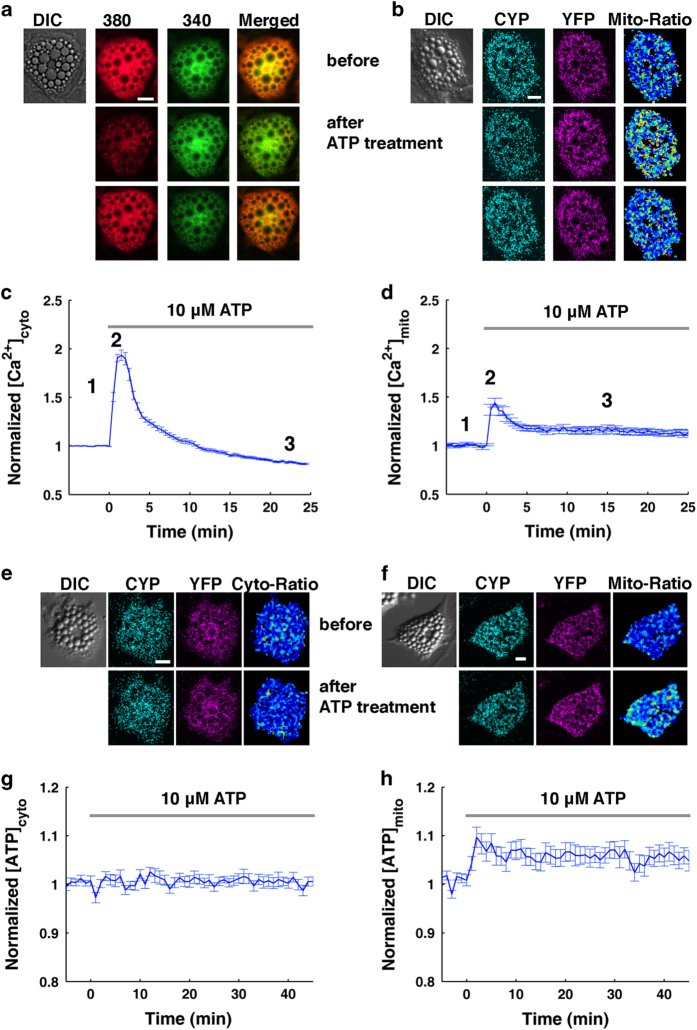
Sympathetic cotransmitter ATP enhances NE-induced thermogenic efficacy in BA by modulating the function of mitochondrial complex V. (**a**–**d**) Sympathetic cotransmitter ATP increases cytoplasmic and mitochondrial [Ca^2+^] in BA, which are both fluorescence-based ratiometric measurements. Scale bars, 20 μm. (**a**) Shows fluorescence images (excited by 380-nm or 340-nm laser) of Fura2-AM-stained BA before and after 10 μM ATP treatment, and positions in **c** are sequentially labeled with 1, 2 and 3. (**b**) Shows fluorescence images of 4mtD3cpv-transfected BA before and after 10 μM ATP treatment, and positions in **d** are sequentially labeled with 1, 2 and 3. (**c** and **d**) Show that ATP induces transient elevations of cytoplasmic (*n*=63) and mitochondrial (*n*=14) [Ca^2+^] in BA, respectively. (**e**–**h**) Fluorescence of AT1.03-based ratiometric measurements of cytoplasmic and mitochondrial ATP concentrations induced by extracellular ATP. (**e** and **f**) Show fluorescence images of AT1.03- and mitAT1.03-transfected BA before and after 10 μM ATP treatment, respectively. Scale bars, 20 μm. (**g** and **h**) Show the averaged change of cytoplasmic (*n*=28) and mitochondrial (*n*=31) ATP by 10 μM ATP treatment. All data points in **c**, **d**, **g** and **h** represent mean±s.e.m.

To elucidate the enhancement effect of sympathetic cotransmitter ATP via purinergic receptors, we evaluated the statuses of cytoplasmic and mitochondrial Ca^2+^ concentrations ([Ca^2+^]) with ratiometric probe Fura2-AM and indicator 4mtD3cpv,^27^ respectively (Figure 4 and Supplementary Figure 4). We found that extracellular ATP evokes a transient elevation of cytoplasmic [Ca^2+^] (Figures 4a and c) in BA through multiple P2 receptors (Supplementary Figure 7 and Supplementary Movie 5), which is in line with previous reports.^28^ In contrast, we found that extracellular ATP evoked transient peaked and steady elevation of mitochondrial [Ca^2+^] (Figures 4b and d).

Mitochondrial [Ca^2+^] is well recognized as an important control of cellular ATP homeostasis.^29^ Therefore, we examined the states of cytoplasmic and mitochondrial [ATP] after ATP stimulation (Figure 4e–h). We observed a steady elevation of mitochondrial [ATP] and no apparent change of cytoplasmic [ATP] after extracellular ATP stimulation (Figures 4e–h). These findings suggest that sympathetic cotransmitter ATP increases the ATP synthase activity of complex V for ATP production (Figure 4h) by elevating mitochondrial [Ca^2+^] (Figure 4d). Importantly, the thermogenic enhancing function (Figure 3) of ATP as a sympathetic cotransmitter of NE can explain the discrepancy of cold and sympathomimetics activities on human BAT *in vivo*.^30^

## Discussion

### Mitochondrial complex V is a deterministic factor in NE-induced thermogenic efficacy

How does the H^+^-ATPase activity of complex V modulate the thermogenic efficacy of BA? A first straightforward explanation is the endothermic characteristic feature of ATP hydrolysis by mitochondrial complex V,^31^ which pumps dissipated protons back and restores energy in the mitochondrial proton gradient. In Figure 1e, there are some interesting and supporting results, where very few BA show endothermic profiles rather than exothermic profiles in response to NE stimulation. The endothermic profiles could be a stimulus artifact, but this outcome is unlikely. Second, the H^+^-ATPase activity of complex V results in cytoplasmic acidification (Figures 1i and 2h), which can enhance the binding and inhibition of purine nucleotides to UCP1.^32^

Why does complex V need to function as an H^+^-ATPase by NE stimulation? The physiological role of an H^+^-ATPase function is likely to avoid unnecessary and undesired heat production as NE is either secreted by adrenal medullae or released by the sympathetic nervous system, which can also be excited by mental or physical stress.^33^ Accordingly, for cold stimulation, our observations suggest that at least two factors (NE and ATP) are needed for the high efficacy and specificity of evoking thermogenesis in BAT. Compared with NE+oligomycin or CCCP-induced fully thermogenic responses (Supplementary Figure 6), NE+ATP can utilize ~60% of the full capacity (Figure 3).

In this study, we observed that NE evokes both homogenous responses (redox ratio, cytosol ATP consumption and calcium dynamics) and heterogeneous changes (ΔΨ_m_, ΔpH and thermogenesis). Consequently, we have demonstrated that heterogeneous changes and thermogenic efficacy are modulated by the functional status of mitochondrial complex V and sympathetic transmitters. NE-induced heterogenic responses in BA suggest that it may use a frequency-modulating method for NE-induced mild thermogenesis (~25% of thermogenic capacity, Figure 1h). NE co-stimulation with ATP or oligomycin A can evoke thermogenesis in BA with both frequency and amplitude modulations (Figure 3 and Supplementary Figure 6), which are the net results of proton outflow by the H^+^-ATPase of mitochondrial complex V and proton leakage by UCP1.

Our findings suggest that mitochondrial malfunctions or impairments in the sympathetic system or BAT can be pathological reasons for inefficient energy expenditure and the development of obesity. Additionally, our current observations also open new doors and pave the way to reconsider targets and strategies that partially or fully utilize the thermogenic capacity of brown or browning^34^ adipocyte for the treatment of obesity, diabetes and abnormal lipid metabolism.
