# Supplementary material for: Sympathetic transmitters control thermogenic efficacy of brown adipocytes by modulating mitochondrial complex V
Source: Signal Transduct Target Ther. 2017 Nov 10;2:17060–. doi: 10.1038/sigtrans.2017.60 (PMC5680398; doi:10.1038/sigtrans.2017.60)
Supplement: Supplementary Information [file sigtrans201760-s1.pdf]

Supplemental Data

Sympathetic Transmitters Control Thermogenic Efficacy of Brown Adipocytes by Modulating Mitochondrial Complex V

Tao-Rong Xie1*, Chun-Feng Liu1*,2, Jian-Sheng Kang1#

1. CAS Key Laboratory of Nutrition and Metabolism, Institute for Nutritional Sciences, Shanghai Institutes for Biological Sciences, Chinese Academy of Sciences; University of Chinese Academy of Sciences, P. R. China.
2. Technical Center for Animal, Plant and Food Inspection and Quarantine, Shanghai Entry-Exit Inspection and Quarantine Bureau, P. R. China

Supplemental Data include seven supplemental figures, five supplemental movies and supplemental materials.

Supplemental Data include seven supplemental figures, five supplemental movies and supplemental materials.

1. Supplemental figures and legends

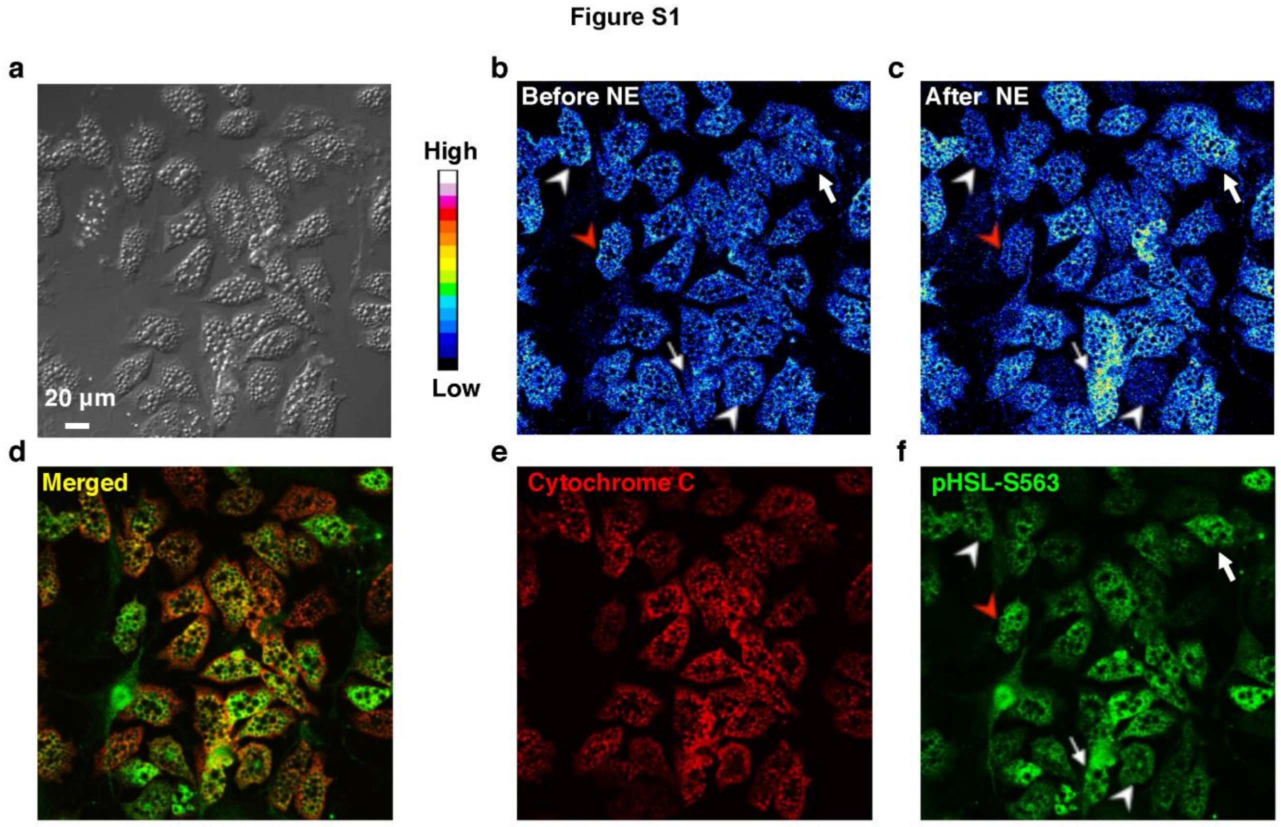

Figure S1. NE-induced heterogeneous changes of mitochondrial membrane potentials (MMP) are not correlated to the heterogeneity of phospho-HSL levels. **(a-c),** Merged confocal images of mitochondria in BA stained with 20 nM mitochondrial marker Rh800. Scale bar, 20 µm. **(a),** Differential interference contrast (DIC) image of multilocular BA. (**b** and **c),** Representative images of mitochondrial membrane potential (represented with Rh800 intensity) are shown for the moments before and after 0.1 µM NE treatment respectively. Arrow heads point to BA with mitochondrial depolarization, while arrows point to BA with mitochondrial hyperpolarization. (**d-f),** Heterogeneous changes of MMP in BA are not correlated with phosphorylated levels of HSL. Retrospectively immunostained images of BA after NE treatment. **(d),** Merged image of BA stained with antibodies against cytochrome C (red, **e**) and phosphorylated HSL (green, **f**). Red arrow head points to BA with mitochondrial depolarization (**b** and **c**) and high level of phosphorylated HSL (**f**), while white arrows point to BA with mitochondrial hyperpolarization (**b** and **c**) and high level of phosphorylated HSL (**f**). Meanwhile, arrow heads show BA with mitochondrial depolarization (**c**) and low level of phosphorylated HSL (**f**).

Figure S2

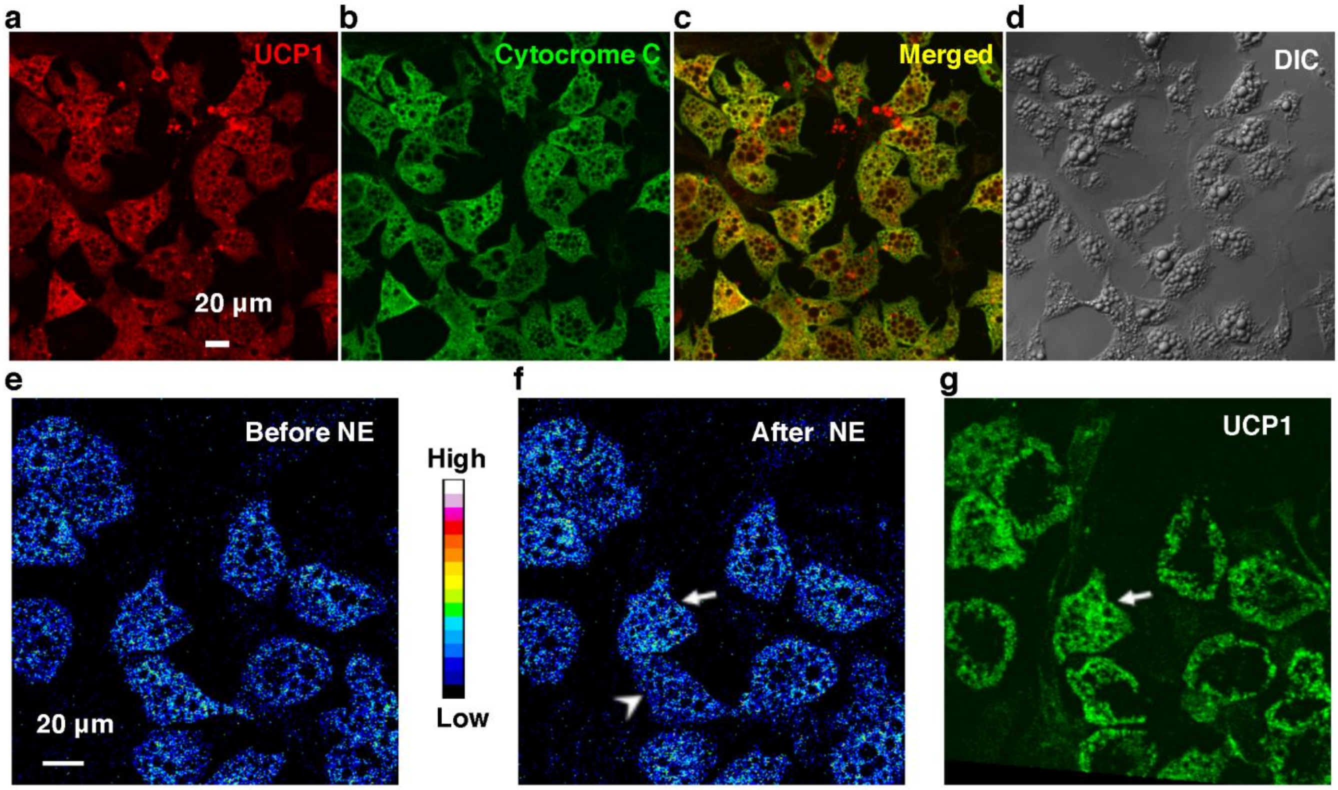

Figure S2. Hyperpolarization of MMP in BA is not due to the absence or heterogeneity of UCP1 levels. (a-d), Confocal images of BA immunostained with antibodies against UCP1 (red, **a**) and Cytochrome C (green, **b**). Scale bar, 20 µm. **c,** Merged image of immunostained BA. (**d**)**,** Differential interference contrast (DIC) image of BA. (**e** and **f**)**,** Hyperpolarization of MMP in BA is not correlated with expression levels of UCP1. Representative images of mitochondrial membrane potential (represented with Rh800 intensity) are shown for the moments before and after 0.1 µM NE treatment respectively. (**g**)**,** Confocal images of BA retrospectively immunostained with antibody against UCP1 after NE treatment (**e** and **f**). Arrow points to BA with mitochondrial hyperpolarization with high level of UCP1, while arrow head shows BA with mitochondrial depolarization.

Figure S3

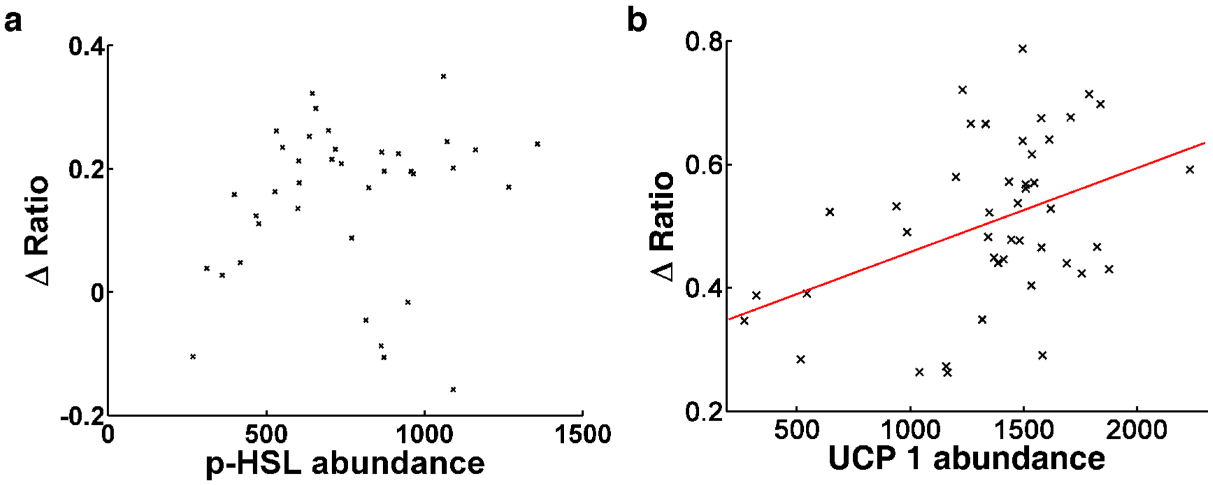

Figure S3. Relationships among thermogenic responses, p-HSL and UCP 1 in BA after NE treatment.

(**a**)**,** Thermogenic ratio versus the abundance of p-HSL in NE-treated BA, which shows little correlation (r = 0.16, n = 38, P = 0.3308, by Pearson's correlation coefficient test). Each black cross represents a paired value (p-HSL versus thermal ratio) of single BA. (**b**)**,** Thermogenic ratio versus the abundance of UCP1 expression in NE-treated BA, which shows a moderate and positive correlation (red line, r = 0.41, n = 44, P = 0.0052, by Pearson's correlation coefficient test). Each black cross represents a paired value (UCP1 versus thermal ratio) of single BA.

Figure S4

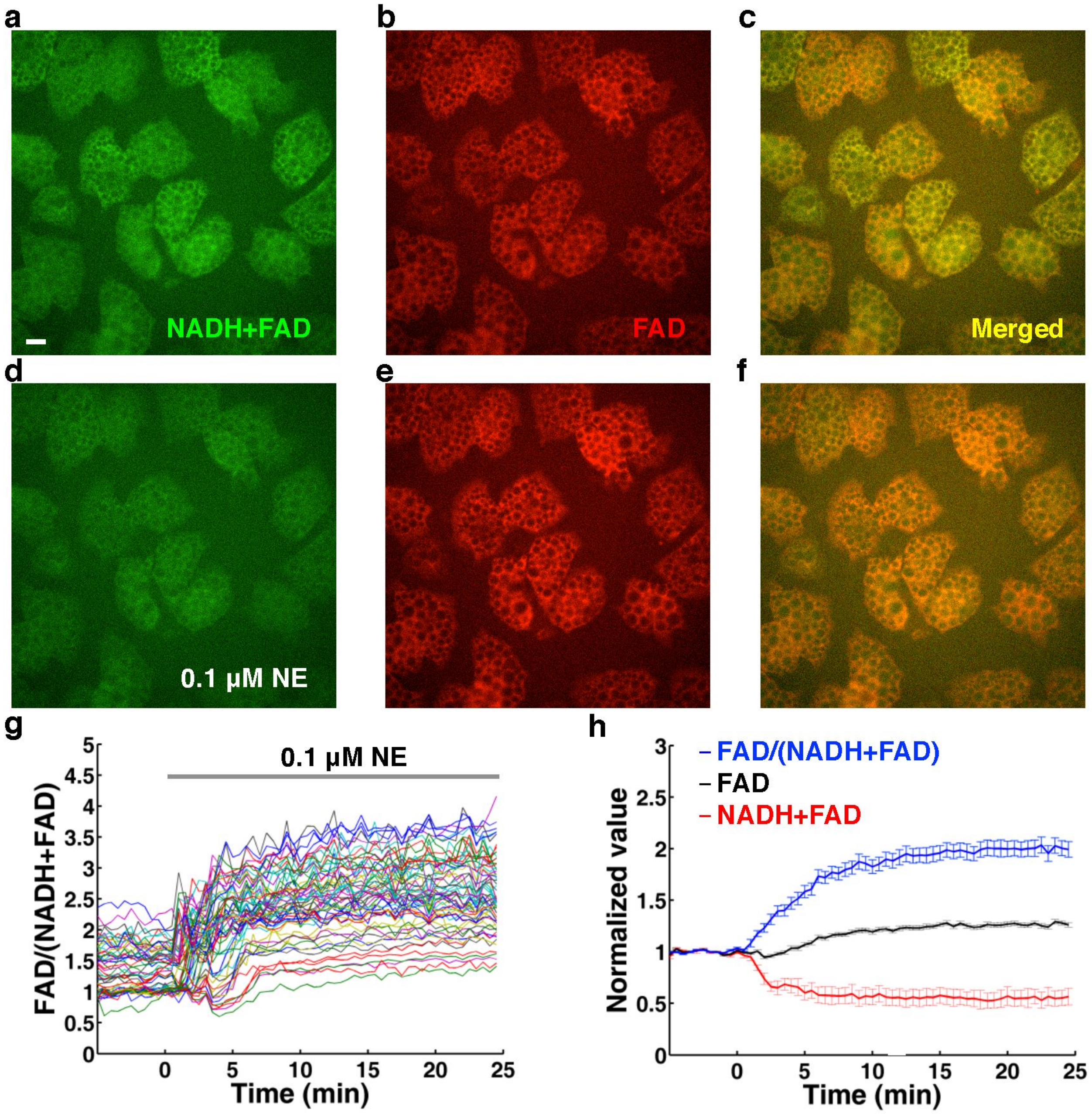

Figure S4. Redox ratio measurement for NE stimulation in BA. (a-h), Autofluorescence of FAD and NADH based redox ratio measurements. Scale bar, 20 μm. (**a** and **d**), show autofluorescence images of FAD + NADH signals before and after 0.1 μM NE treatment respectively, while (**b** and **e**) show autofluorescence images of FAD before and after NE treatment respectively. (**c** and **f**) are merged redox ratio images of FAD (red) to FAD+NADH (green). (**g**), The raw data plots of redox ratio change in 0.1 μM NE treated BA. Each colored trace represents redox change of single BA (n = 60). **(h)** shows the normalized averaged FAD, FAD+NADH and redox change after NE treatment. All data points in (**h**) represent mean ± SEM.

Figure S5

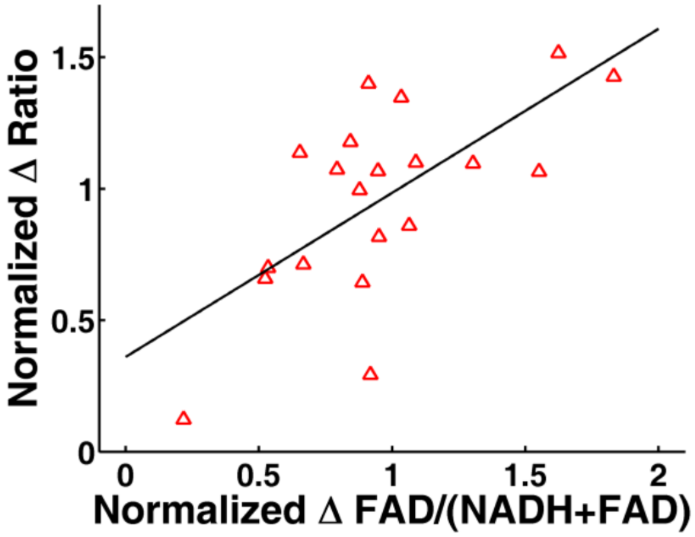

Figure S5. Depolarization of MMP and thermogenic response in oligomycin-pretreated BA after NE treatment.

A representative plot of thermogenic ratio versus redox ratio in NE-treated BA, which shows a strong and positive correlation (black line, r = 0.66, n = 20, P = 0.0014, by Pearson's correlation coefficient test). Each red hollow triangle represents a paired value (redox ratio versus thermal ratio) of single BA.

Figure S6

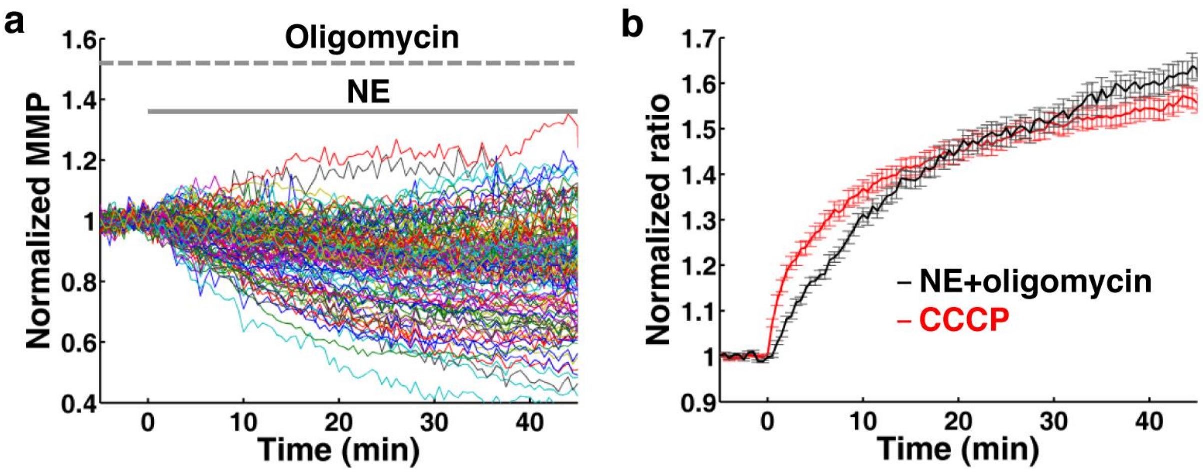

Figure S6. Depolarization of MMP and thermogenic response in oligomycin-pretreated BA after NE treatment.

(a) Representative raw data plots of MMP show NE-induced depolarization in oligomycin-pretreated BA. Each colored trace represents a MMP change of single BA (n = 113). **(b)** shows that 0.1 μM NE-induced thermogenic responses (black, n = 113) in oligomycin-pretreated BA is comparable to 10 μM CCCP-induced thermogenic responses (red, n = 88, data reused from ref 10). All data points in figures represent mean ± s.e.m.

Figure S7

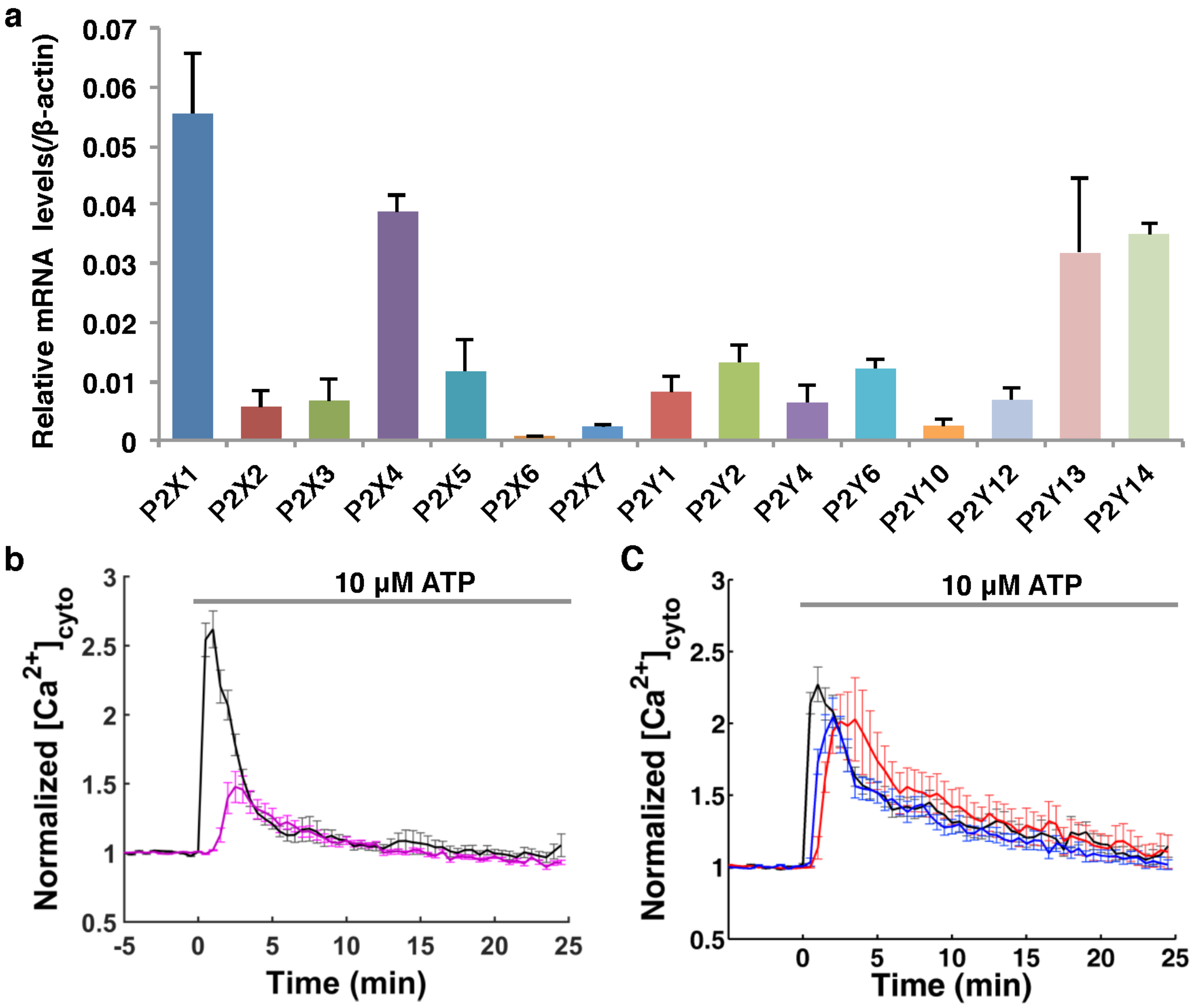

Figure S7. Sympathetic cotransmitter ATP increases intracellular [Ca^{2+}] in BA through multiple P2 receptors. (a), The quantitative-PCR results show the expression levels of P2 receptors in BA, including P2X1, P2X2, P2X3, P2X4, P2X5, P2X6, P2X7, P2Y1, P2Y2, P2Y4, P2Y6, P2Y10, P2Y12, P2Y13 and P2Y14. (**b** and **c),** show that ATP induces cytoplasmic [Ca^{2+}] transient in BA can be dramatically decreased with multiple inhibitors of P2 receptors **(b)**, and less affected by single inhibitor of P2X receptor (**c**). **(b)** shows that the combination of multiple inhibitors (10 μM PPADS, a nonselective inhibitor of P2X receptor; 2.5 μM PSB 0739, an antagonist of P2Y12; 0.5 μM MRS 2279, an inhibitor of P2Y1; 100 μM Suramin, a nonselective inhibitor of P2Y receptor) can largely decrease the cytoplasmic [Ca^{2+}] transient induced by ATP stimulation (Control, black, n = 25; multiple inhibitors, magenta, n = 21). **(c)** shows that single inhibitor has little effect to reduce cytoplasmic [Ca^{2+}] transient induced by ATP stimulation (Control, black, n = 17), such as a selective antagonist of P2X1 (10 μM PPNDS, blue, n = 14) or nonselective inhibitor of P2X receptor (10 μM PPADS, red, n = 13). All data points in **a-c** represent mean ± SEM.

2. Supplemental Movie Legends

Supplemental Movie 1 | Subpopulations of NE-induced thermogenesis in BA. The ratiometric pseudocolor movie of 0.1 μM NE-induced thermal responses in BA. Scale bar, 20 μm.

Supplemental Movie 2 | NE-induced heterogeneous responses in BA. The raw channel data of supplementary movie 1 show BA subpopulations after NE treatment. Red represents the channel of thermosensitive RhB-ME, while green represents thermoneutrol Rh800. Scale bar, 20 μm.

Supplemental Movie 3 | NE and ATP co-induced thermogenesis in BA. The ratiometric pseudocolor movie of co-treatment with 0.1 μM NE and 10 μM ATP-induced thermal responses in BA. Scale bar, 20 μm.

Supplemental Movie 4 | NE and ATP co-induced responses in BA. The raw channel data of supplementary movie 3 show BA subpopulations after NE and ATP co-treatment. Red represents the channel of thermosensitive RhB-ME, while green represents thermoneutrol Rh800. Scale bar, 20 μm.

Supplemental Movie 5 | ATP-induced cytoplasmic [Ca^{2+}] transient in BA. The merged pseudocolor movie of 10 μM ATP-induced cytoplasmic [Ca^{2+}] transient in BA. Scale bar, 20 μm.

3. Supplemental Materials

General materials

Rhodamine B (RhB), Rhodamine 800 (Rh800), and carbonyl cyanide m-chlorophenyl hydrazone (CCCP), rotenone, oligomycin A, adenosine triphosphate (ATP), anti-UCP1 (U6382, rabbit), collagenase type II, cytosine arabinoside (Ara-C), Ficoll 400, glutathione, glucose, Hepes, KCl, NaCl, $MgCl_2$ and $CaCl_2$ were purchased from Sigma-Aldrich Corporation (USA). Norepinephrine (NE) and Dimethyl Sulfoxide (DMSO) were purchased from Santa Cruz Biotechnology, Inc. (USA). SR-59230A was purchased from Abcam Inc. (UK). SNARF1-AM, Fura2-AM, Dulbecco's Modified Eagle Medium (DMEM), fetal bovine serum (FBS), newborn calf serum (NCS), ALEXA-488 goat anti-rabbit, ALEXA-555 goat anti-mouse, Phosphate-Buffered Salines, pH7.4 (PBS) and Penicillin Streptomycin (Pen Strep) were purchased from Thermo Fisher Scientific Co., Ltd. (USA). Anti-Phospho-HSL (Ser563, 4139, rabbit) was purchased from Cell Signaling Technology (USA). Matrigel and anti-cytocrome C (556432, mouse) were purchased from BD Biosciences Company (USA). Coverslips were purchased from Glaswarenfabrik Karl Hecht GmbH & Co KG (Germany).

C57BL/6J mice were purchased from Sino-British SIPPR/B&K Lab Animal Ltd., Shanghai (China). All experimental procedures and protocols were approved by the Institutional Animal Care and Use Committee of the Institute for Nutritional Sciences, Shanghai Institutes for Biological Sciences, Chinese Academy of Science.

All confocal imaging was performed using confocal microscope FW1000 (Olympus Corporation, Japan). The other fluorescence imagings were performed using a customized fluorescence microscope (BX61WI, Olympus Corporation, Japan) equipped with Optoscan monochromator (Cairn Research Ltd., UK), customized emission filters (Semrock, USA) and Evolve 512 EMCCD (Photometrics Ltd., USA), which were all controlled with customized Micro-Manager software.

Primers for quantitative-PCR analysis of P2 receptors in BA

	Forward primers	Reverse primers
Actin	5’-TTCTTTGCAGCTCCTTCG-3’	5’-TTCTGACCCATTCCCACC-3’
P2x1	5’-GACAAACCGTCGTCACCTCT-3’	5’-ATCCCAGAGCCGATGGTAGT-3’
P2x2	5’-CCACCACCACTCGAACTCTC-3’	5’-CAGAGCAGTGGCCAGATTGA-3’
P2x3	5’-TCCTGAAGGCTTTCGGCATC-3’	5’-CACTCCCACGGAAGTGAAGG-3’
P2x4	5’-CCCAGATATTCCTTCCGGCG-3’	5’-GTGAGTGTGCGTTGCTCATT-3’
P2x5	5’-GCCAGGTATTACCGTGACCC-3’	5’-CTGAATTTTCCCGCCTTGCC-3’
P2x6	5’-ACCCAGGTTAAGGAGCTGGA-3’	5’-TGGTGTCACGAGGAAGTTGG-3’
P2x7	5’-GGGGTGACGAAGTTAGGACA-3’	5’-ACTTGGCCTTCTGACTTGACA-3’
P2y1	5’-CCAATGTGCCCTGACCAAGA-3’	5’-ACATCCAGATAGCCACGCTG-3’
P2y2	5’-TCCTATGCCGCCTCAAAACC-3’	5’-TAAATGGCCAGTGGTCACCC-3’
P2y4	5’-CCAACCCTTTGGCTGTTCCT-3’	5’-GGGCAGTGACAGCACATACA-3’

P2y6	5’-GGGTAGTGTGTGGAGTCGTG-3’	5’-GCGAGTAGACAGGATGGGTG-3’
P2y10	5’-ACTGCCTGTTTGCCATTTCC-3’	5’-CAACCATGGTGACCAGAACCA-3’
P2y12	5’-GTTCCCTTCCACTTTGCACG-3’	5’-AGGGTGCTCTCCTTCACGTA-3’
P2y13	5’-CCGTCTGGTCCCTGATGTTC-3’	5’-GCCCAAGGGGACTCTTCAAA-3’
P2y14	5’-CCACATTGCCAGAATCCCCT-3’	5’-AGCCGAGAGTAGCAGAGTGA-3’

Total RNA of cultured brown adipocytes were extracted and prepared with Trizol (Thermo Fisher, Cat No: 15596-026). First strand cDNAs were synthesised using oligo(dT)18 primers (Thermo Fisher, First Strand cDNA Synthesis Kit, Cat No: K1612) and RT-PCR reagent, which contains 4.6 µl cDNA (100 ng/µl), 0.2 µl each primer (10 µM) and 5 µl SYBR green mix (Thermo Fisher, Cat No: 4367659). The paired primers of *P2x* and *P2y* were listed as below:

All the reagents were pipetted into 384-well plate, gently spun down, and placed in 7500 Real-Time PCR System (ABI). Samples were Incubated at 95°C for 10 min, followed by 40 cycles of 95°C for 25 s, 60°C for 30 s, and 72°C for 30 s. Data were collected and analyzed by being normalized with actin.
